# The Largest Known Survival Analysis of Patients with Brain Metastasis from Thyroid Cancer Based on Prognostic Groups

**DOI:** 10.1371/journal.pone.0154739

**Published:** 2016-04-29

**Authors:** Jinhyun Choi, Jun Won Kim, Yo Sup Keum, Ik Jae Lee

**Affiliations:** 1 Department of Radiation Oncology, Gangnam Severance Hospital, Yonsei University College of Medicine, Seoul, Korea; 2 Department of Radiation Oncology, Yonsei Cancer Center, Yonsei University College of Medicine, Seoul, Korea; 3 Texas A&M Health Science Center College of Medicine, Bryan, Texas, United States of America; Van Andel Institute, UNITED STATES

## Abstract

**Purpose:**

To analyze the clinical features and prognostic factors associated with the survival of patients with a very rare occurrence of brain metastasis (BM) from differentiated thyroid cancer (DTC).

**Methods and Materials:**

A total of 37 patients with DTC who were diagnosed with BM between 1995 and 2014 were included. We reviewed the clinical characteristics, treatment modalities, and image findings of BM. Factors associated with survival were evaluated, and the patients were divided into three prognostic groups (Groups A, B, and C) for comparative analysis.

**Results:**

The median age at BM was 63 years, and the median time from initial thyroid cancer diagnosis to BM was 3.8 years. The median survival and the 1-year actuarial survival rate after BM were 8.8 months and 47%, respectively. According to univariate and multivariate analyses, four good prognostic factors (GPFs) were identified including age ≤ 60 years, PS ≤ ECOG 2, ≤ 3 BM sites, and without extracranial metastasis prior to BM. Three prognostic groups were designed based on age and number of remaining GPFs: patients ≤ 60 years of age with at least 2 GPFs (Group A) had the most favorable prognosis with a median survival of 32.8 months; patients ≤ 60 years of age with fewer than 2 GPFs and those > 60 years of age with at least 2 GPFs (Group B) had an intermediate prognosis with a median survival of 9.4 months; and patients > 60 years of age with fewer than 2 GPFs (Group C) had the least favorable prognosis with a median survival of 1.5 months.

**Conclusions:**

The survival of patients with BM form DTC differed among the prognostic groups based on the total number of good prognostic factors.

## Introduction

Carcinoma of the thyroid gland is the most common cancer, accounting for 16.6% of all newly diagnosed cancers in Korea [1]. The incidence of thyroid cancer is increasing worldwide, particularly because of a rise in papillary thyroid cancer, the most common form of thyroid cancer. The prognosis of thyroid cancer is related to the histology subtype and the stage at the time of diagnosis. Thyroid cancer is usually indolent with good prognosis and long-term survival, even with its high incidence of distant metastases [2–4]. The overall incidence of distant metastases varies between 10% and 35%, depending upon the histology [3].

The major sites of distant metastases from thyroid cancer are the lung (70%) and bone (20%), and multiple sites are involved in 10–20% of patients at the time of diagnosis [5]. Although patients with M1 thyroid cancer might live for a prolonged period with the disease, the presence of distant metastasis has a significant impact on patient survival [6]. Meanwhile, thyroid cancer represents a brain metastasis (BM) from approximately 1% of all differentiated thyroid cancer (DTC) to around 10% of anaplastic patients. The reported median survival of patients diagnosed with BM varies between 4 months and 33 months [5, 7, 8]. Because of its rarity, the impact of BM on the survival of patients with DTC is unknown, and there are no clear guidelines for managing patients with BM from DTC. For BM in general, although treatment guidelines can differ based on the prognosis of the patients and the extent of the BM, surgery remains the preferred treatment modality [9]. With the indolent course and rarity of BM from DTC, however, more information on treatment outcomes is needed to support the standard guidelines for BM management.

In this study, we report the clinical features and potential prognostic factors associated with the survival of patients with a very rare occurrence BM from DTC.

## Materials and Methods

### Patients

Between 1995 and 2014, a total of 37 histologically confirmed DTC patients were diagnosed with BM at out institution. This Institutional Review Board of the Severance Hospital, Korea (IRB No. 3-2015-0134) approved this retrospective study in accordance with ethical guidelines and the Declaration of Helsinki. The consent was not necessary, because patient records and information were anonymized and de-identified prior to analysis. We retrospectively reviewed the medical records of the patients, including the clinical presentation, treatment received, radiologic features of BM, and clinical course. All of the reviewed patients had undergone surgery for primary thyroid cancer, and the pathology was confirmed. When BM was clinically suspected, the patients underwent computed tomography (CT) scans or magnetic resonance imaging (MRI) to confirm the diagnosis of BM. We examined the following clinical factors for association with survival: age, sex, time interval between initial thyroid cancer diagnosis and BM, Eastern Cooperative Oncology Group (ECOG) performance status (PS), presenting symptoms of BM, treatment modality for BM, number of brain lesions, tumor size, extracranial metastases diagnosed prior to BM (present or absent), and primary thyroid cancer recurrence. Among them, factors showed a statistically potential association with survival defined as good prognostic factors (GPFs). Based on these factors, we divided the patients into three prognostic groups according to the total number of potential prognostic factors established in the analysis. Survival curves among the prognostic groups (Groups A, B, and C) were compared. In cases of radiosurgery, both Gamma Knife and linear accelerator-based therapies were included.

### Statistical analysis

Overall survival was defined from the date of BM diagnosis to the date of the last follow-up or death. Survival data were analyzed using the Kaplan-Meier method. The number of BM lesions was counted on imaging. The tumor size was measured as the longest diameter of the largest intracranial tumor regardless of the number and location of the brain lesions. A univariate analysis was performed using the log-rank test to identify prognostic factors associated with survival, and multivariate analyses were performed using the Cox proportional hazards model. Statistical analysis was carried out using SPSS version 20 (SPSS Inc., Chicago, USA). P values ≤ 0.05 were considered statistically significant.

## Results

### Patient and tumor characteristics

The characteristics of the patients and tumors are presented in Table 1. The median age of the patients was 59 (range, 29–76) years at initial diagnosis of thyroid cancer and 63 (range, 39–78) years at BM diagnosis. The median time interval from diagnosis of thyroid cancer to BM development was 3.8 (range, 0–30.7) years. Twenty-one patients were female, and 16 patients were male. The histological subtypes were 32 papillary thyroid carcinoma, 3 follicular carcinoma, and 2 poorly differentiated carcinoma. The ECOG PS at BM diagnosis was 0–1 (16 patients), 2 (14 patients), or 3 (7 patients). Twenty-five patients were diagnosed with BM based on neurologic symptoms including headache, nausea, mental change, ataxia, and motor or sensory deficits, and 12 patients without symptoms were diagnosed with BM incidentally during evaluation for their initial or synchronous multiple distant metastases. The most common symptoms were motor deficits followed by headache and dizziness. The site of first presentation of distant metastasis was the lung for 25 patients and the brain for 13 patients, and 4 patients had both lung and brain metastases simultaneously. The median interval between initial lung metastasis and subsequent BM was 12.9 months. At the time of BM diagnosis, 21 patients had recurrent primary thyroid tumors, and 24 patients had previously diagnosed extracranial metastases.

**Table 1:** Clinical characteristics of the 37 patients with brain metastasis from thyroid cancer.

Characteristics	No. of patients (%)
**Age at diagnosis of brain metastasis (years)**	
**Median (range)**	63 (39–78)
**Time from diagnosis of thyroid cancer to BM (years)**	
**Median (range)**	3.8 (0–30.7)
**Sex**	
**Male**	16 (43.2)
**Female**	21 (56.8)
**Histology**	
**Papillary**	32 (86.5)
**Follicular**	3 (8.1)
**Poorly differentiated**	2 (5.4)
**Performance status**	
**ECOG 0–1**	16 (43.2)
**ECOG 2**	14 (37.8)
**ECOG 3**	7 (18.9)
**Symptoms**	
**Sensory deficit**	1 (2.7)
**Motor deficit**	8 (21.6)
**Headache**	4 (10.8)
**Nausea/vomiting**	2 (5.4)
**Mental change**	3 (8.1)
**Ataxia**	2 (5.4)
**Dizziness**	4 (10.8)
**Memory loss**	1 (2.7)
**None**	12 (32.4)
**Number of BM sites**	
**1–3**	21 (56.8)
**4–9**	11 (29.7)
**≥ 10**	5 (13.5)
**Site of initial distant metastasis**	
**Lung**	25 (67.6)
**Brain**	13 (35.1)
**Lymph node**	2 (5.4)
**Bone**	4 (10.8)
**Spine**	5 (13.5)
**Other**	3 (8.1)

### Radiologic findings of BM and treatment profiles

All of the patients performed CT (51.3%), MRI (89.2%), or both (40.5%) for BM diagnosis. The study included 9 patients with 1 BM site, 12 patients had 2 or 3 BM sites, 11 patients had from 4 to 9 BM sites, and 5 patients had more than 10 BM sites. The mean value of maximum metastatic tumor diameter was 2.1 cm.

Metastases were treated by surgical resection alone in 3 patients (1–5 lesions), radiosurgery alone in 8 patients (≥1 lesions), partial-brain radiotherapy (PBRT) in 6 patients (1–4 lesions) and whole-brain RT (WBRT) in 6 patients (≥3 lesions). Three patients underwent resection followed by WBRT for 1–4 lesions, and one patient underwent resection followed by PBRT for a single lesion. Three patients received radiosurgery combined with RT: two underwent WBRT, and one underwent PBRT after post-radiosurgery progression. Seven patients including one who received chemotherapy were managed with conservative care without surgery or RT. Among the 19 patients who received RT, 9 underwent PBRT, and 10 underwent WBRT. The RT schedule was 37.5–71.25 Gy in 10–30 fractions for PBRT and 25–40 Gy in 8–15 fractions for WBRT. Most of the radiosurgery was performed by Gamma Knife with a maximal dose of 30 Gy.

### Survival and prognostic subgroups

The median survival after BM was 8.8 (range, 0.7–109) months. The overall survival at 1 year and 2 years was 47% and 30.2%, respectively. The median survival was 16.7 months for the BM-treatment group (n = 30) and 2.6 months for the non-treatment group (n = 7; p < 0.001). In the univariate analysis, four factors showed a significant association with survival. Age had a strong impact on survival; the median overall survival was 32.8 months for patients ≤ 60 years of age versus 8.8 months for patients > 60 years of age (p = 0.002). Patients with PS ≤ ECOG 2 had a median survival of 16.7 months, whereas patients with PS > ECOG 2 had a median survival of 1.6 months (p < 0.001). The median survival of patients with 1–3 BM sites was 30.7 months, whereas that of patients with more than 3 BM sites was only 8.8 months (p = 0.004). With respect to the treatment modality, the median survival was better for patients who underwent either surgical resection or radiosurgery (30.7 months) compared with that for patients who did not undergo those treatment modalities (5 months; p = 0.001). The median survival among patients with extracranial metastases prior to BM diagnosis (10 months) was worse than that among patients without prior extracranial metastases (19.2 months; p = 0.065). There was no statistically significant effect on survival of sex, time interval between initial thyroid cancer diagnosis and BM diagnosis, presence of neurologic symptoms, history of radiotherapy (RT), or primary site recurrence. The age (HR = 9.146, p = 0.004), PS (HR = 4.449, p = 0.031), number of BM sites (HR = 3.455, p = 0.016), and extracranial metastasis prior to BM (HR = 4.829, p = 0.005) were independent prognostic factors for survival in the multivariate analyses. According to these results, four GPFs were identified including age ≤ 60 years, PS ≤ ECOG 2, ≤ 3 BM sites, and without extracranial metastasis prior to BM. The results of the univariate and multivariate analyses are summarized in Table 2.

**Table 2:** Univariate and multivariate analysis of predictors associated with survival in patients with brain metastasis from thyroid cancer.

variable	n	Univariate			Multivariate	
		median (mon)	95% CI	*p*	HR	*p*
**Age (years)**				0.002		0.004
**≤ 60**	15	32.8	5.5–60		Ref.	
**> 60**	22	8.8	5.4–12.1		9.146	
**Sex**				0.266		
**Male**	16	19.2	8.1–30.2			
**Female**	21	10	7.7–12.2			
**Time interval (years)**				0.634		
**≤ 3**	18	10	7.7–12.2			
**> 3**	19	19.4	4.3–34.4			
**ECOG PS**				<0.001		0.031
**≤ 2**	30	16.7	6.6–26.8		Ref.	
**> 3**	7	1.6	0.0–3.2		4.449	
**Neurologic symptoms**				0.571		
**Yes**	25	11.5	5.1–17.8			
**No**	12	11.7	0.0–25.9			
**Radiotherapy**				0.901		
**Yes**	19	11.7	3.3–20.0			
**No**	18	11.5	3.7–19.2			
**OP or SRS**				0.001		0.092
**Yes**	18	30.7	3.0–58.3		Ref.	
**No**	19	5	0.0–11.3		2.658	
**Number of BM sites**				0.004		0.016
**≤ 3**	21	30.7	8.8–52.5		Ref.	
**> 3**	16	8.8	2.2–15.3		3.455	
**Prior DM**				0.065		0.005
**Yes**	24	10	5.4–14.5		4.829	
**No**	13	19.2	6.9–31.4		Ref.	
**Primary site recurrence**				0.248		
**Yes**	21	11.5	6.6–16.3			
**No**	16	11.7	2.6–20.7			

Abbreviations: PS = performance status; OP = operation; SRS = stereotactic radiosurgery; BM = brain metastasis; DM = distant metastasis

**Fig 1:**
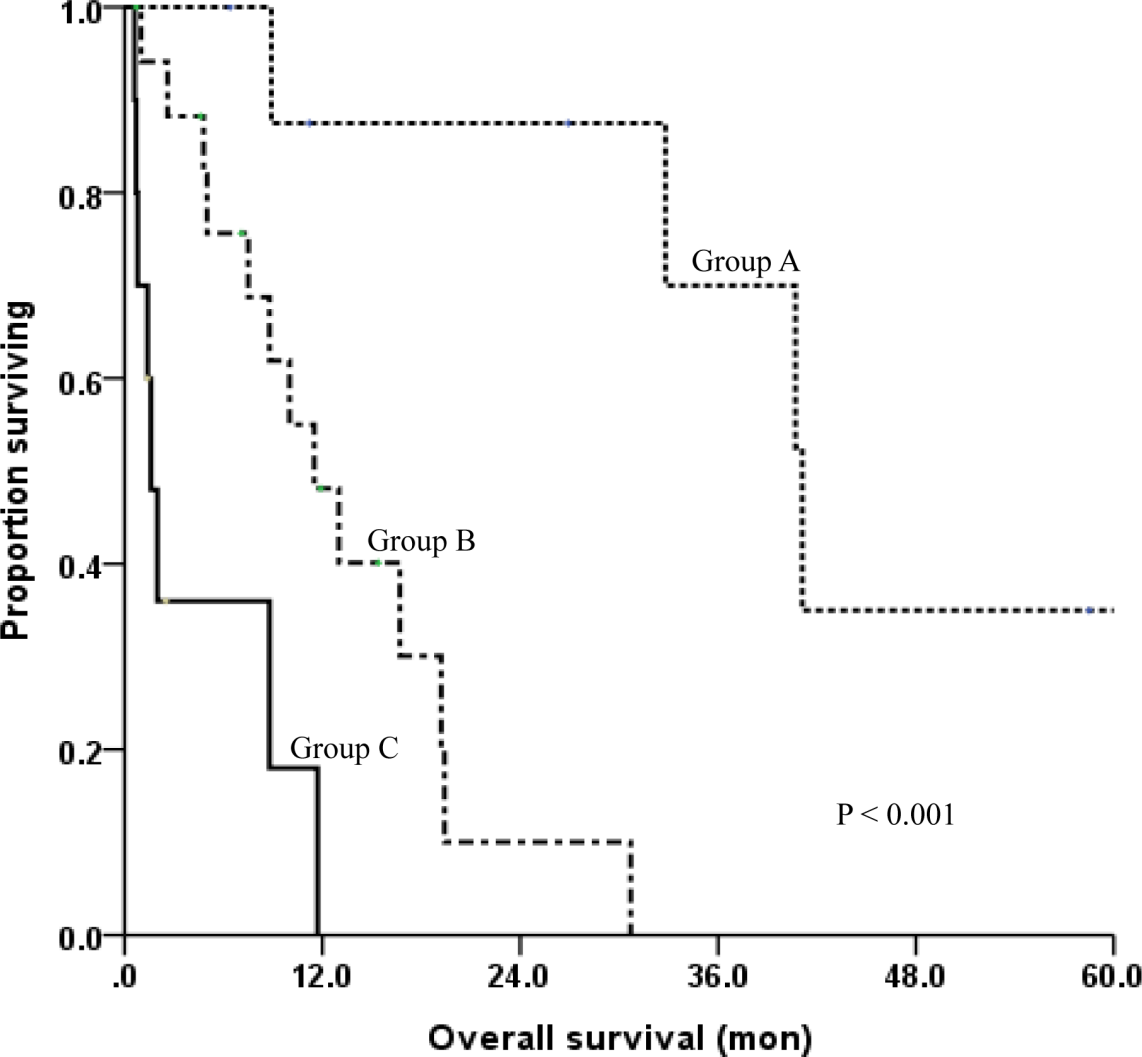
Overall survival comparison among the three prognostic subgroups. Group A: patients ≤ 60 years of age and at least 2 good prognostic factors (GPF); Group B: patients ≤ 60 years of age and fewer than 2 GPF or > 60 years of age with at least 2 GPF; Group C: patients > 60 years of age and fewer than 2 GPF.

Based on the independent prognostic factors excluding treatment modality (surgery or radiosurgery), the patients were divided into 3 prognostic groups: patients ≤ 60 years of age with at least 2 GPFs (Group A); patients ≤ 60 years of age with fewer than 2 GPFs and those > 60 years of age with at least 2 GPFs (Group B); and patients > 60 years of age with fewer than 2 GPFs (Group C). As shown in Table 3, the survival rates differed significantly among the prognostic groups. Group A (n = 9) had a median survival of 32.8 months and a 1-year survival rate of 87.5%, while Group C (n = 10), the least favorable group, had a median survival of only 1.5 months and 1-year survival rate of 0%. Group B (n = 18), the intermediate group, had a median survival of 9.4 months and a 1-year survival rate of 48.1%. The survival curves of the three prognostic groups are compared in Fig 1.

**Table 3:** Comparison of overall survival rates among prognostic subgroups.

	Prognostic subgroup		
	Group A (n = 9)	Group B (n = 18)	Group C (n = 10)
**Definition**	Age ≤ 60 & GPF ≥ 2	Age ≤ 60 & GPF < 2 or Age > 60 & GPF ≥ 2	Age > 60 & GPF < 2
**Survival rates**			
**Median (months)**	32.8	9.4	1.5
**1-year (%)**	87.5	48.1	0

Abbreviations: GPF = good prognostic factor, including performance status ≤ ECOG 2, ≤ 3 BM sites, and without extracranial metastasis prior to BM

### Treatment patterns and causes of death among the subgroups

The features and outcomes of the nine patients in Group A are described in Table 4. Among those patients, seven received either surgical resection or radiosurgery, and the remaining two received RT alone. The patient with the most favorable outcome had all of the GPF and was treated with radiosurgery for a single BM lesion. No evidence of further disease progression was found in that patient during the last follow-up. Three patients in Group A had only short-term survival (11.2, 6.4, and 8.9 months), however, resulting from follow-up loss or the treatment of multiple BM. No patient in Group A died from a brain progression. On the other hand, 5 of the 10 patients in Group C were managed only with conservative treatment, and 3 of those patients died due to brain progression.

**Table 4:** Features and outcomes of nine patients belonging to Group A.

Patient	Sex	Age at BM (years)	Histology	Time interval from TC to BM (years)	Performance status (ECOG)	Number of BM	Neurologic symptoms	Treatment for BM	Extracranial metastasis	Current status	OS (months)
**1**	M	58	papillary	7.6	2	1	motor deficit	Op+3D-CRT	lung	AWD	11.2
**2**	F	50	papillary	10.2	1	2	headache	IMRT	lung	AWD	6.4
**3**	M	50	papillary	4.1	1	2	none	Radiosurgery	lung	DOD	40.7
**4**	F	50	papillary	3.8	1	3	headache, nausea	Radiosurgery	lung	DOD	32.8
**5**	M	50	papillary	4.1	1	2	none	Radiosurgery	none	DOD	41.1
**6**	F	55	papillary	synchronous	2	≥10	motor deficit	Radiosurgery+WBRT	none	DOD	8.9
**7**	M	42	papillary	synchronous	0	1	none	Radiosurgery	none	NED	109
**8**	F	54	follicular	synchronous	1	1	ataxia, headache	Op+WBRT	none	AWD	58.5
**9**	M	49	follicular	synchronous	2	1	headache, dizziness	IMRT	none	AWD	26.9

Abbreviations: BM = brain metastasis; TC = thyroid cancer; DM = distant metastasis; OS = overall survival; OP = operation; CRT = conformal radiotherapy; IMRT = intensity modulated radiotherapy; WBRT = whole-brain RT; AWD = alive with disease; DOD = death of disease; NED = no evidence of disease

## Discussion

Several studies have investigated the treatment outcomes of BM from primary cancers including lung, breast, and colorectal cancers and melanoma. In breast cancer, the second most frequent cause of BM, the BM typically occurs in the late stages of metastatic breast cancer. In one study, the median overall survival after BM from breast cancer was 11.5 months, and while the treatment outcome differed among cancer subtypes (ER, PR, Her2 status), focal brain treatment could be considered for better local control of BM in patients with aggressive subtypes [10]. BM from hepatocellular carcinoma (HCC) is frequently associated with intracranial hemorrhage, because HCC is hypervascular, and most patients have coagulopathy. Although the overall prognosis for patients with BM from HCC is extremely poor with median survival of only 6.8 weeks, some subsets of patients manifest favorable survival criteria including single brain metastasis and good liver function [11].

The clinical courses and outcomes of BM from thyroid cancer are unclear, because the prevalence of BM from DTC is rare. In this study, we investigated the clinical features and prognostic factors of 37 patients with BM from DTC. The most frequent site of first distant metastasis was the lung followed by the brain. Most of the patients died with lung progression, although the patients who were treated for BM had a better survival compared to those who were not. Also, considering that the median time interval from the initial diagnosis of thyroid cancer to the diagnosis of BM was 3.8 years, both the BM and the primary cancer follow an indolent course, suggesting that aggressive treatment of both intracranial and extracranial metastases is needed for longer survival. Although BM tended to be identified more commonly in patients with aggressive histologic types, BM originating from poorly differentiated thyroid carcinoma was extremely rare in this study. It is in line with previously published reports that included mostly patients with DTC. [5, 12–14]

The management of BM from DTC remains undetermined because of a paucity of recent data, and most of the published reports are based on data from old series with symptomatic patients [5]. It is widely thought that chemotherapy is seldom helpful for most type of thyroid cancer and distant metastasis from thyroid cancer, which may support the non-chemotherapy treatments of BM implemented in this study. General treatment guidelines can be differentiated based on the prognosis of the patients and the extent of the BM disease [10]. Maximal surgical resection followed by stereotactic radiosurgery (SRS) or WBRT results in better survival rates compared with WBRT alone [15, 16]. The use of radiosurgery is increasing for the treatment of a limited number of brain metastases. SRS alone or in addition to WBRT has been shown to achieve excellent local control rates and patient functional status [17]. Therefore, surgical resection or radiosurgery might have been performed in patients with one or a limited number of BM lesions, an absent or a controlled systemic disease, a life expectancy ≥ 3 months, and a good PS [18, 19]. For decades, WBRT has been the standard treatment for patients with multiple lesions, a life expectancy < 3 months, or a low Karnofsky Performance Status (KPS) score [18, 19]. Those guidelines are not specific, however, to patients with thyroid cancer. In this study, the median survival after BM was 8.8 months, but the patients who were treated with surgery or radiosurgery had a median survival of 30.7 months compared with only 5 months for those who did not receive surgery or radiosurgery. Chie et al. [20] reported that among 36 patients with BM from thyroid cancer, those who received surgery or SRS had a median survival of 16.7 months, whereas those who did not receive those treatments had a median survival of 4.7 months. Another single-institutional study showed that overall survival among patients who received surgery or SRS was 37.4 months compared with 20.8 months among patients who did not receive those treatments [21]. Unfortunately, we could not find the effect of even local RT on patient survival in our study.

We believe that this study is the largest among the published reports evaluating the treatment outcomes of BM from DTC. We evaluated the treatment outcomes based on three prognostic groups. Prognostic factors that help predict patient survival are important for making treatment decisions for individual patients. There are at present several prognostic indices to guide treatment decisions for patients with BM [22–24]. The Radiation Therapy Oncology Group (RTOG) recursive partitioning analysis (RPA) [22], which is the index most commonly used to evaluate BM prognosis, includes 3 classes: Class I includes patients < 65 years of age with KPS ≥ 70, controlled primary tumor, and no extracranial metastases; Class III includes patients with KPS < 70; and Class II includes all patients not in Classes I or III. In order to incorporate the number of metastases and eliminate treatment factors for the guidance of treatment choice, the Graded Prognostic Assessment [25] was developed, which sums up the scores (0, 0.5, and 1.0) for each of the 4 prognostic factors (age, KPS, extracranial metastases, and number of central nervous system metastases). Despite the fact that those indices were derived through the analysis of many possible prognostic factors, the biological behaviors of various primary tumors can be quite different, making it difficult to apply one common index to all types of BM. Rades et al. [26] included independent predictors of survival (gender, KPS, and extracranial metastases) in a scoring system for BM from non-small cell lung cancer, in which the score for each of the factors was obtained from the 6-month survival rate divided by 10. The survival rate was very different among the three prognostic groups defined based on the total score, providing a tool to help offer the best available treatment to each patient. With respect to thyroid cancer prognosis, which is related to the histologic type, the treatment outcomes for patients with BM could differ from those for patients with other primary cancers. Furthermore, we found that BM from thyroid cancer had a radioresistant feature, so survival estimation could help determine the appropriate treatment. Consequently, it seems useful to group patients with BM from thyroid cancer according to their expected survival. Because age was the strongest prognostic factor for both BM and primary thyroid cancer, three prognostic groups were designed based on a number of other prognostic factors including PS, number of BM sites, and distant metastasis prior to DM diagnosis. Patients in Group A had the most favorable prognosis with a median survival of 32.8 months. Meanwhile, patients in Group B had an intermediate prognosis with a median survival of 9.4 months, and patients in Group C had the least favorable prognosis with a median survival of only 1.5 months. The survival was thus very different among the three groups. Based on those group classifications, we can estimate the expected survival of patients with BM from DTC before we treat the BM. We found that some specific groups of patients could expect long-term survival over 30 months as a result of appropriate treatment. Although this study reflects the treatment results of only a small group of patients, we can expect improved outcomes with surgical resection or radiosurgery, especially for patients classified in Group A with oligometastases of brain.

This study was a retrospective review of previously collected data, and there might have been a selection bias with respect to the treatment modality received, because the patients who received either surgery or radiosurgery were very specifically selected. The majority of the patients who received WBRT underwent only palliative treatment for multiple BMs and had a poor PS, the factors that had little impact on survival. In addition, the prognostic subgroups defined in the current study have not been validated in a prospective study, which is not likely to be expected because of the rare incidence of BM from DTC.

## Conclusion

The results of this retrospective study show that clinical features such as age (≤ 60 years), PS (≤ 2), number of BM sites (≤ 3), and absence of previous distant metastasis prior to BM development were independently associated with survival. Survival differed among the prognostic groups based on those clinical features. Therefore, we can estimate the survival of patients with BM from DTC based on those prognostic factors and recommend more definitive treatment such as surgery or radiosurgery for long-term survival in patients with GPF.
